# Molecular Mapping and Validation of a Major QTL Conferring Resistance to a Defoliating Isolate of Verticillium Wilt in Cotton (*Gossypium hirsutum* L.)

**DOI:** 10.1371/journal.pone.0096226

**Published:** 2014-04-29

**Authors:** Xingju Zhang, Yanchao Yuan, Ze Wei, Xian Guo, Yuping Guo, Suqing Zhang, Junsheng Zhao, Guihua Zhang, Xianliang Song, Xuezhen Sun

**Affiliations:** 1 State Key Laboratory of Crop Biology/Agronomy College, Shandong Agricultural University, Taian, Shandong, China; 2 Cotton Research Center, Shandong Academy of Agricultural Sciences, Jinan, Shandong, China; 3 Heze Academy of Agricultual Sciences, Heze, Shandong, China; USDA-ARS-SRRC, United States of America

## Abstract

Verticillium wilt (VW) caused by *Verticillium dahliae* Kleb is one of the most destructive diseases of cotton. Development and use of a VW resistant variety is the most practical and effective way to manage this disease. Identification of highly resistant genes/QTL and the underlining genetic architecture is a prerequisite for developing a VW resistant variety. A major QTL *qVW-c6-1* conferring resistance to the defoliating isolate V991 was identified on chromosome 6 in LHB22×JM11 F_2∶3_ population inoculated and grown in a greenhouse. This QTL was further validated in the LHB22×NNG F_2∶3_ population that was evaluated in an artificial disease nursery of V991 for two years and in its subsequent F_4_ population grown in a field severely infested by V991. The allele conferring resistance within the QTL *qVW-c6-1* region originated from parent LHB22 and could explain 23.1–27.1% of phenotypic variation. Another resistance QTL *qVW-c21-1* originated from the susceptible parent JM11 was mapped on chromosome 21, explaining 14.44% of phenotypic variation. The resistance QTL reported herein provides a useful tool for breeding a cotton variety with enhanced resistance to VW.

## Introduction

Cotton (*Gossypium* spp.) is the most important fiber crop, and the second most important source for edible oil and protein in the world [1]. Cotton production is affected by both biotic and abiotic stresses, of which Verticillium wilt (VW) caused by soil-borne *Verticillium dahliae* Kleb is one of the most destructive diseases in cotton. VW was first reported in Virginia, US in 1914 [2] and is found in almost all cotton growing areas worldwide. The infected plants usually exhibit symptoms of marginal chlorosis or necrosis in leaves, discoloration of the stem vascular bundles, even full defolation and plant death. Severe infection results in significant reduction in plant mass, lint yield and fiber quality [3], [4]. There were two major VW outbreaks in China, one was in 1993 and the other in 2002–2003, each resulting in about 100,000 tons of lint yield loss [5]. There are no effective solutions to control this disease once a plant is infected [6]. Thus, VW is called cancer of cotton in China.

The VW has not been effectively controlled mainly due to its biology characteristics, the indeterminacy of the genetic mechanism of resistance to VW and the lack of highly resistant commercial Upland cotton varieties, except for some modern Acala cotton cultivars developed in California and New Mexico [7]. *V. dahliae* has a wide host range of over 400 plant species including herbaceous annuals and perennials and woody perennials [8], and can survive in soil for many years in the dormant form of microsclerotia or as mycelium or conidia in the vascular system of perennial plants. These properties make crop rotation, even field fallowing less effective. Although application of soil fumigants, such as methyl bromide, is an effective control strategy, but expensive and harmful to both environment and human health [9]. Planting a resistant or tolerant cultivar has long been considered as the most practical, economic and effective means of decreasing lose from VW.

As for VW resistance heredity, two different genetic models, qualitative trait model and quantitative trait model, were reported by researches using different materials in traditional genetic studies [10]. With the development and utilization of molecular marker technologies, much progress in QTL mapping for VW resistance in cotton has been achieved which provides more information about the genetics of VW resistance at molecular level. So far, more than 100 QTL conferring VW resistance have been mapped on twenty-two chromosomes (Chr.) of tetraploid cotton except for Chr.6, 10, 12 and 18 from interspecific populations of *G. hirsutum*×*G. barbadense* [3], [11]–[17] and from *G. hirsutum* intraspecific populations [18]–[21] evaluated at different growing stages with different *V. dahliae* isolates. However, comparison of these results indicated that VW resistance was characterized as specific to both *V. dahliae* isolates and plant growth stages [15], [16], [21], with some exceptions where chromosomal regions with broad-spectrum VW resistance on Chr.16, 23 in *G. hirsutum* germplasm 60182 [18] and on Chr.23 in *G. hirsutum* varieties Prema [19] were identified, respectively. Previous studies also indicated that the resistance QTL number varied largely from 1∼3 [13], [14], [21] to 14 [18] with cotton varieties/lines studied even for one *V. hahliae isolate*, implying there be differences in genetic basis for VW resistance in different materials. More recently, a large number of genes were identified to express differentially in defense responses to different races (V991 and D07038) of *V. dahliae* through cDNA-AFLP [22], RNA-sequencing [23], [24] and proteomic and virus-induced gene silencing [25]. These researches indicated that genes that involved in the lignin, gossypol, brassinosteroids, jasmonic acid and the phenylalanine metabolism play important role in cotton defense responses to different races of *V. dahliae*. Several important clues regarding VW resistance have been obtained, including Bet v1 and UbI gene family in *G. hirsutum* cv. Zhongzhimian KV1 [22], lignin-synthesis related genes in *G. barbadense* cv. Hai7124 [24], the phenylalanine metabolism related genes in Zhongzhimian KV1 and *G. barbadense* cv. Xinhai 15 [23], and genes involved in gossypol, brassinosteroids and jasmonic acid in Hai7124 [25].

The defense responses to VW infection are also associated with some other factors, such as the phonological stage of the plant, environmental conditions, level of inoculum, and the virulence of *V. dahliae* strains [26]. So integrated resistance evaluation using local isolates both in greenhouse for seedling stage resistance and in disease nursery and/or naturally infected field for adult stage resistance was suggested to get reliable disease evaluation results in cotton breeding.

Our previous studies indicated LHB22 possessed high resistance to VW at seedling stage under greenhouse conditions [27] and at mature stage under artificially infected nursery conditions [28]. The objective of this study was to reassess the VW resistance of LHB22 at different growth stages under controlled (greenhouse and artificially infected disease nursery) and naturally infected field environments, and to identify QTL of VW resistance from LHB22, so as to research the genetic architecture of VW resistance and provide molecular tool for cotton breeding program.

## Materials and Methods

### Plant materials

Three variety/germplasm lines were chosen for this research. LHB22 (Lu Hirsutum-Bickii 22), characterized by pink petals and filaments, with a large purplish red spot in the petal base introgressed from *G. bickii* [27], [29], exhibits high resistance to VW [27], [28], and has been widely used as a parent in hybrid development and breeding programs in China. Jimian 11 (JM11), a susceptible control variety in national VW resistance evaluation test of China was provided by the Cotton Research Institute, Chinese Academy of Agricultural Sciences (CAAS). Nannonggan (NNG) introduced from Nanjing Agricultural University was also used as a susceptible parent in this study to map VW resistance in different genetic backgrounds.

In winter of 2008, LHB22 and JM11 were crossed at Sanya breeding station of Shandong province (SYBS/SDP), Hainan, China. In 2009, the subsequent F_1_ seeds were planted and self-pollinated to produce F_2_ progeny in the breeding field of Boyang Seed Company, Huimin, Shandong, China. The parental and F_2_ seeds were then planted at SYBS/SDS in winter of 2009 and self-pollinated to produce 243 F_2∶3_ families (designated as LHB22×JM11 population) used for VW resistance evaluation. In order to validate the QTL located on Chr.6 detected in LHB22×JM11 population, another F_2∶3_ population of 226 families (named as LHB22×NNG) using LHB22 and NNG as parents was constructed with the same methodology in 2009 and 2010.

### VW resistance assay under greenhouse conditions

A defoliating *V. dahliae* isolate, V991, which is prevailing in the Yellow and the Yangtze River cotton growing regions [19] was used for disease infection. This isolate was kindly provided by the Institute of Plant Protection, CAAS, Beijing, China. It was grown on agar petri dish plates for 7–10 days. Then, the isolate mass was increased by growing the isolate on boiled and sterilized cotton seeds in conical flasks for 12–15 days at 25°C. After adding 200 ml of sterile water, the flasks were shaken at 60–100 rpm for 10 minutes to produce a high concentrated conidial suspension which was filtered through 4-layers of cheesecloth, and the conidial concentration was determined with a hemacytometer and adjusted to 1×10^7^ conidia ml^−1^. Conidia suspension was prepared immediately before being used for inoculation.

VW resistance evaluation of LHB22×JM11 population was conducted in a greenhouse at Crop Research Station of Shandong Agricultural University (CRS/SDAU), Taian, China. On September 18 2010, acid-delinted seeds were planted in 10-cm-diameter pots with 2 seeds per pot and 14 pots per family in each replication. The experiment was arranged in a randomized completed block design with two replications for F_2∶3_ families and three replications for two parents. The pots were filled with sterilized potting mixture (sand: peat: clay loam  =  1∶1∶1, vol: vol). At two-true leaf stage, the pot bottom was gently torn off with scissors, each pot was placed in one petri dish containing 10 ml of *V. dahliae* conidia suspension. After the conidia suspension was sucked dry, the pots/plants were planted in a seedling bed in the greenhouse. The plants were irrigated once a week. The average temperature was 24–27°C in the daytime and 18–20°C at nighttime.

Twenty-five days after inoculation, phenotyping of VW resistance was conducted using a severity rating system ranging from 0 to 4 for leaf disease [19], where 0  =  healthy plant without disease symptom; 1  =  one to two yellowish cotyledons; 2  =  two yellowish cotyledons and one symptomatic true leaf or one cotyledon abscised; 3  =  yellowish cotyledons and two symptomatic true leaves, or two cotyledon abscised and one symptomatic true leaf; 4  =  complete defoliation or dead plant. Mean value of all plants was calculated as the score of each F_2∶3_ family in a replicate and average values of two replicates were used in analysis.

### VW resistance assay under disease nursery conditions

VW resistance assays of LHB22×NNG population was performed in an artificially infected disease nursery at the CRS/SDAU in 2011 and 2012. The VW disease nursery was inoculated with cotton seed cultured with isolate V991 for three years since 2007 and once every two years since 2010, resulting in a relatively severe and uniform soil infection by VW. Seeds of plant materials were acid-delinted before use. All F_2∶3_ families were planted in two fully randomized replications with three replications of two parents evenly distributed among them. Each plot had two rows of 5.0 m long in 2011 and one row of 8.0 m long in 2012. The row spacing and plant spacing were 50 cm and 15 cm respectively in two years. The planting date was April 27 in 2011 and April 30 in 2012.

VW resistance was evaluated at early flowering stage on July 15 only in 2011 and at mature stage on October 6–8 in both 2011 and 2012. The disease scores at early flowering stage in 2012 were not collected due to relatively severe drought unsuitable for VW development. Leaf disease symptoms at early flowering stage were scored with a 5-score system ranging from 0 to 4 used as national standard for screening cotton VW resistance in China [16], [18], [19], where score 0 represents healthy plant without symptoms, score 1 represents <25% chloropic/necrotic leaves, score 2 represents 25–50% chloropic/necrotic leaves, score 3 represents 50–75% chloropic/necrotic leaves, and score 4 represents >75% chloropic/necrotic leaves or complete defoliation and plant death. The vascular tissue symptoms were evaluated by the ratio of the length of symptomatic vascular tissue to the entire plant length [16] with a 5-score system ranging from 0 to 4, where score 0 represents healthy vascular without symptoms, score 1 represents <25% symptomatic vascular area, score 2 represents 25–50% symptomatic vascular area, score 3 represents 50–75% symptomatic vascular area, and score 4 represents >75% symptomatic vascular area. All plants (55–60 plants in 2011 and 45–50 plants in 2012 respectively) in a spot were scored to get spot value and averages of two replications were used in QTL analysis.

### VW resistance assay under naturally infected field conditions

In order to further test the effect of the major resistance QTL *qVW-c6-1* which was detected on Chr.6 at more generations, four bulked F_4_ of the LHB22×NNG population were made through the Graphical GenoTypes software GGT32 (http://www.dpw.wau.nl/pv/pub/ggt/) based on the molecular marker data, including two resistant bulks (BulkR1, BulkR2) and two susceptible bulks (BulkS1, BulkS2). The two resistant bulks contained a homozygous chromosome segment between markers GH433 and DPL0665 from LHB22 (containing the resistance allele of *qVW-c6-1*), while the two susceptible bulks possessed homozygous chromosome segments of the same marker interval from NNG (not containing the resistance allele of *qVW-c6-1*). Each bulk was comprised of equal volume of seeds from 8 different F_2∶3_ families. This experiment was conducted in 2012 in a field severely infected by mixed isolates including V991 of *V. dahliae* in Dezhou, Shandong province, which belongs to the Yellow River cotton growing region of China. The four F_4_ bulks and the two parents (LHB22, NNG), together with another cotton (*G. hirsutum*) variety Yu2067 which is widely used as a resistant control in VW resistance evaluation test in China, were planted on April 26 in a randomized complete block design with three replications. Each replicate had two rows of 10 m long. The row space was 80 cm and plant space was 33 cm. Twenty plants in the middle of each plot were used for VW resistance evaluation. VW resistance was scored at flowering stage (July 8) and at mature stage (October 7) with the same methodology as described above.

### DNA isolation and SSR assays

Genomic DNA was isolated from young leaves from each F_2_ plant of the two populations and the three parental varieties using the CTAB method [30]. The SSR technique was used to identify polymorphic markers for construction of a genetic linkage map. Mainly based on two previously reported maps [31], [32], a total of 5400 SSR primers were used to screen polymorphisms between LHB22 and JM11. PCR amplifications were conducted on a Peltier Thermal Cycler (M J Research) using the PCR reaction mixture of a previous work [33] following the reaction program: 95°C for 2 min; 30 cycles of 94°C for 45 s, 57°C for 45 s and 72°C for 60 s; and a final extension at 72°C for 10 min. SSR primers were obtained from the Cotton Marker Database (CMD) (http://www.cottonmarker.org). PCR products were separated and visualized using sodium dodecyl sulfate polyacrylamide gel electrophoresis and silver staining method [34].

### Map construction and QTL mapping

Genetic maps were constructed using JoinMap 3.0 [35]. A minimum LOD value of 4.0 and a recombination frequency of 0.40 were used as thresholds to identify linkage groups. Marker distances in centi Morgan (cM) were estimated with the Kosambi mapping function [36]. Linkage groups were assigned to chromosomes by bridge loci with existing consensus maps of tetraploid cotton [31], [32], [37]. QTL detection was performed by composite interval mapping (CIM) using Windows QTL Cartographer 2.5 (http://statgen.ncsu.edu/qtlcart/WQTLCart.htm). A stringent LOD threshold through 1000-permutation test was utilized to determine significant QTL. The percentage of the phenotypic variation explained by a QTL was estimated at the highest probability peak.

### Data analysis

Data analysis was performed with SPSS18 software. For the bulked F_4_ assay, a one-way ANOVA was conducted and the least significant difference (LSD) was used to verify the differences between the varieties or genotypes at p = 0.05. Pearson correlation coefficients between disease severity scores at two stages were also calculated.

## Results

### VW resistance evaluation

**Figure 1:**
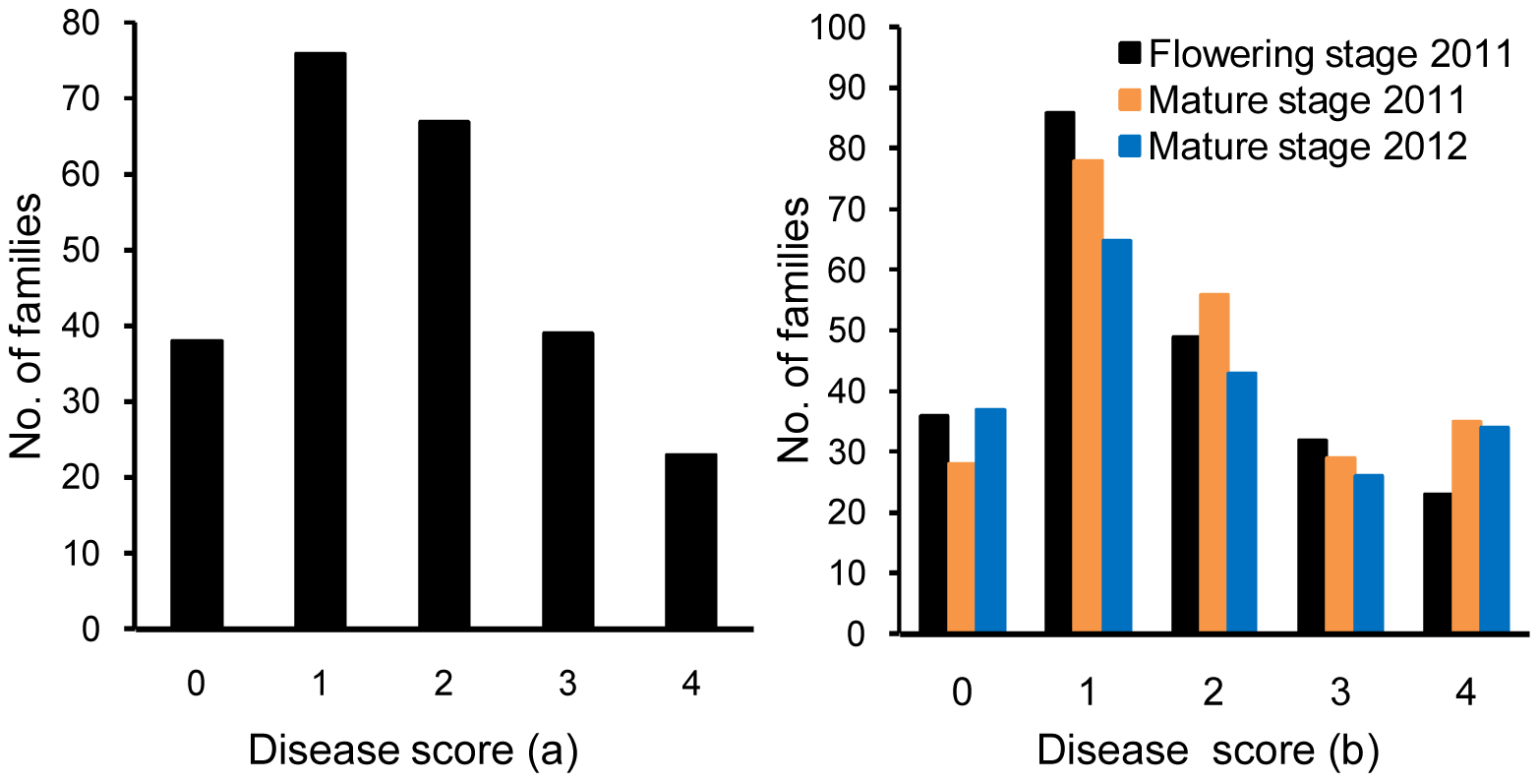
The frequency distribution of disease scores. (a) LHB22×JM11 F_2:3_ population was screened at seedling stage in greenhouse in 2010. (b) LHB22×NNG F_2:3_ population was evaluated in Verticillium wilt disease nursery at two stages in 2011 and 2012.

Plant disease scores of both parents and hybrid progeny (F_1_ and F_2∶3_) families were assayed for leaf symptoms at seedling stage and vascular symptoms at mature stage (Table 1). In greenhouse, both LHB22 and LHB2×JM11 F_1_ showed high resistance (low disease scores) to the isolate V991 at seedling stage, whereas the susceptible JM11 exhibited significant higher disease scores. In the disease nursery, LHB22 and LHB2×JM11 F_1_ showed higher resistance (0.89–1.09; 1.03–1.25) than the susceptible parent NNG (3.41–3.56). Although the disease scores of the two F_1_s were higher than LHB22 both in greenhouse and disease nursery evaluations, the differences were not statistically significant. These data suggested a possible dominant inheritance pattern of LHB22’s VW resistance. Continuous and normal distribution of disease scores was observed in both F_2∶3_ populations grown in a greenhouse and disease nursery at different stages (Figure 1). A significant positive correlation (r = 0.867, *p* = 0.0078) was observed between early flowering stage resistance and mature stage resistance in LHB22×NNG F_2∶3_ population in 2011.

**Table 1:** Average disease severity scores of parents and populations.

	Average disease severity scores			
	Seedling stage	Flowering stage	Mature stage	
Parents and populations	Greenhouse (2010)	Nursery (2011)	Nursery (2011)	Nursery (2012)
LHB22	0.65 ^aA^ ‡			
JM11	3.38 ^bB^			
F_1_	0.77 ^aA^			
F_2:3_	2.45			
LHB22		0.89 ^aA^	1.04 ^aA^	1.09 ^aA^
NNG		3.41 ^bB^	3.56 ^bB^	3.47 ^bB^
F_1_		1.03 ^aA^	1.25 ^aA^	1.21 ^aA^
F_2:3_		2.65	2.77	2.58

‡ Differences among parents and F_1_ of a population were verified by the LSD analysis, and different lowercase letter and capital letter in the same column show difference significant at 0.05 and 0.01 level respectively.

### Linkage map construction

Of the 5400 SSR primer pairs screened, 186 (3.4%) revealed polymorphism between parents LHB22 and JM11. These 186 polymorphic markers were used to genotype the F_2_ population in order to construct a genetic map. Nine markers did not produce clear and stable PCR products among the population, and were discarded. Of the 177 markers analyzed, a total of 182 segregating loci were scored. Of them, 13 exhibited significant deviation from expected Mendelian segregation ratios for 1∶2∶1 or 3∶1 in chi-square tests, and were excluded when mapping. Finally 141 SSR marker loci were mapped on 26 linkage groups, covering 1143.1 cM with an average distance of 8.11 cM between adjacent markers. According to the consensus maps [31], [32], [37], these 26 linkage groups were assigned to 23 chromosomes. No markers were mapped on Chr.2, 20 and 25. The whole linkage map is available in Figure S1.

### QTL mapping of VW resistance at seedling stage under greenhouse conditions

**Figure 2:**
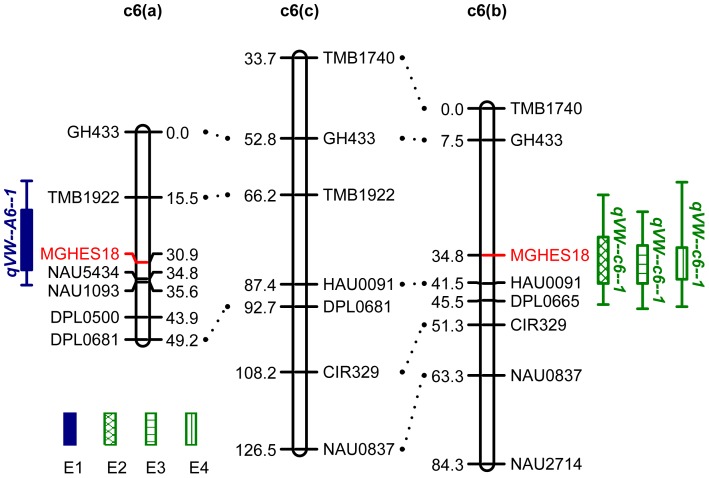
A major QTL conferring resistance to Verticillium wilt detected on chromosome 6. Linkage group c6 (a) was constructed from LHB22×JM11 F_2:3_ population and linkage group c6 (b) was constructed from LHB22×NNG F_2:3_ population. The c6 (c) map was drawn according to a consensus map of cotton [31] and only the related markers were selected. The QTL *qVW-c6-1* was detected in LHB22×JM11 population in 2010 at seedling stage in greenhouse (E1), and was further detected at flowering stage in 2011 (E2) and mature stage in 2011 (E3) and 2012 (E4) in LHB2×NNG F_2:3_ population.

**Figure 3:**
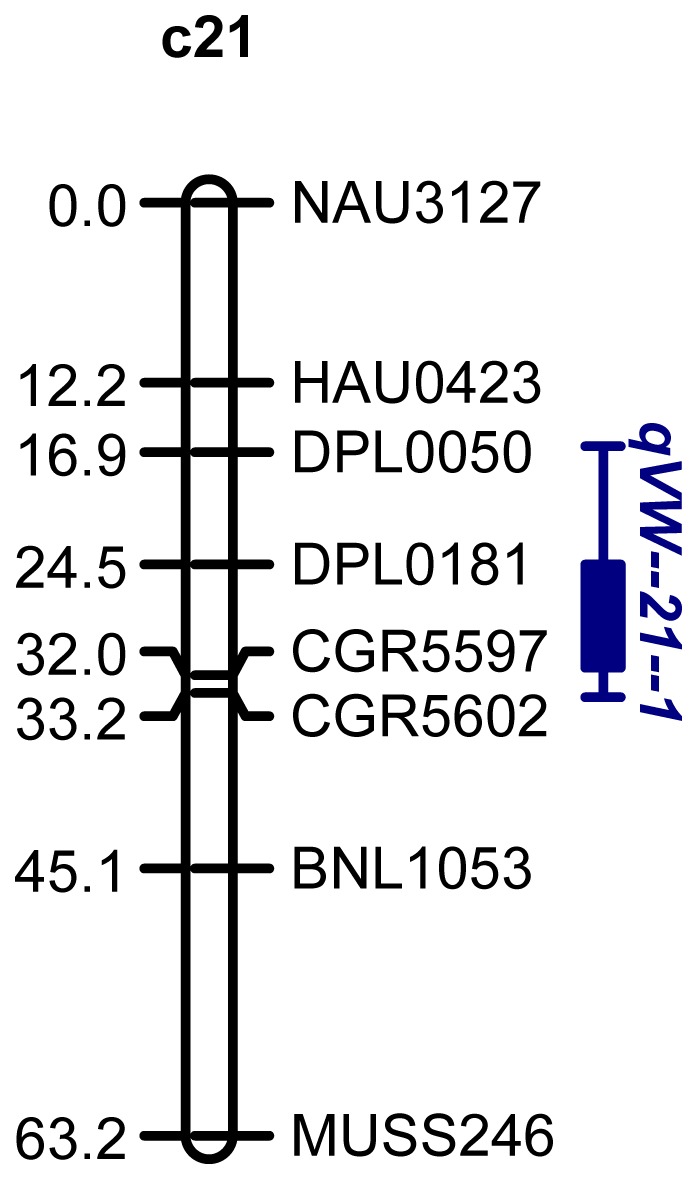
A Verticillium wilt minor resistance QTL detected on chromosome 21 in LHB22×JM11 F_2:3_ population. The susceptible parent JM11 donates the positive allele of this QTL.

Based on a Composite Interval Mapping (CIM) analysis of the disease score data of the LHB22×JM11 population grown in a greenhouse, two VW resistance QTL were identified on Chr.6 and 21. The nearest marker, LOD value, peak position, marker interval, genetic effects and the percent PV explained are summarized in Table 2 and Figure 2, Figure 3). A major resistance QTL, *qVW-c6-1*, explaining 25.90% of the phenotypic variance, was detected tightly linked to MGHES18 on Chr. 6. The additive effect of this QTL could decrease the VW disease score by 0.66. The resistance allele originated from the resistant parent LHB22. The other QTL *qVW-c21-1* was mapped on Chr.21 with a LOD value of 3.4 and could explain 14.44% of the phenotypic variation, whose resistant allele was donated by the susceptible parent JM11. These results also indicated both resistant and susceptible parents may provide resistance QTL for VW resistance improvement in breeding.

**Table 2:** QTL conferring resistance to Verticillium wilt detected in two F_2:3_ populations of *G. hirsutum.*

Population	Growing stage ^a^	QTL	Nearest marker	Position (cM) ^b^	LOD value	Additive effect (a)	Dominant effect (d)	d/a ^c^	Variation explained (%) ^d^	Donor parent ^e^
LHB22×JM11	S (2010)	*qVW-c21-1*	DPL0050	20.6	3.4	0.31	−0.79	2.54	14.44%	JM11
	S (2010)	*qVW-c6-1*	MGHES18	28.8	3.7	−0.66	−0.65	0.98	22.90%	LHB22
LHB22×NNG	F (2011)	*qVW-c6-1*	MGHES18	34.5	4.2	−0.72	−0.78	1.08	25.70%	LHB22
	M (2011)	*qVW-c6-1*	MGHES18	34.5	4.4	−0.88	−0.75	0.85	27.10%	LHB22
	M (2012)	*qVW-c6-1*	MGHES18	34.5	3.9	−0.70	−0.75	1.07	23.10%	LHB22

^a^ Verticillium wilt (VW) resistance of LHB22×JM11and LHB22×NNG population evaluated in a greenhouse and disease nursery, respectively. S =  seedling stage, F =  flowering stage, M =  mature stage.

^b^ Position of peak LOD score.

^c^ Ratio of dominant effect and additive effect.

^d^ Percentage of variance explained at peak LOD score.

^e^ Parent that provided the positive allele of the QTL.

### Verification of the QTL *qVW-c6-1*

In order to verify the major resistance QTL *qVW-c6-1* originated from LHB22 in a different genetic background at different growth stages, another F_2∶3_ population (LHB22×NNG) was developed and evaluated for VW resistance in an artificially infected disease nursery of V991 at early flowering stage and mature stage in two years. As the *qVW-c6-1* was mapped on Chr.6, the seven SSR markers, mapped on Chr.6 in the LHB22×JM11 population, were first used to screen polymorphism between LHB22 and NNG. Only two markers MGHES18 and GH433 were polymorphic. Then 217 SSR primers previously mapped on Chr.6 available at CMD (http://www.cottonmarker.org) and by other works [16], [31], [32], [37]–[39], were selected to screen polymorphism between the two parents. Six polymorphic markers were identified. Finally, a linkage map covering 84.3 cM with 8 SSR markers was constructed (Figure 2) and utilized in QTL analysis with the same methodology mentioned above. One major resistance QTL each for leaf symptom in 2011 and vascular symptom in 2011 and 2012, respectively, was detected on Chr.6 with LOD value 3.9–4.4, explaining 23.1–27.1% of phenotypic variation (Table 2 and Figure 2). As these three QTL shared the same nearest marker MGHES18 and all the positive alleles of these QTL originated from LHB22, it was concluded that they were the same QTL. Although there were only two common markers (GH433 and MGHES18) between the linkage groups of Chr. 6 from the two mapping populations, comparison of QTL positions could be roughly made through a bride consensus map [37] (Figure 2), which suggested that they could be the same QTL. This result also indicated that *qVW-c6-1* could be repeatedly detected in these two genetic backgrounds.

### VW resistance in the bulked F_4_

The VW disease scores in bulked F_4_ families were summarized in Table 3. There were statistically significant differences in disease scores at flowering stage and mature stage between genotypes as determined by one-way ANOVA (F = 131.625–251.082, *p* = 0.008-0.000). Yu2067, LHB22 and the two resistant bulks exhibited significantly lower disease scores than the two susceptible bulks and NNG at early flowering and mature stages. No significant difference in disease scores was detected between Bulk S1 and Bulk S2, or between Bulk R1 and Bulk R2. Although the disease scores of the two resistant bulks and LHB22 were lower than those of Yu2067, the differences among them were not statistically significant except for disease score at mature stage between Bulk R1 and Yu2067. The disease scores at flowering stage was positively correlated with that at mature stage (r = 0.94, *p* = 0.000). These results indicated that the VW resistance of LHB22 was stable from flowering stage to mature stage, and marker-assisted selection of the positive allele of QTL *qVW-c6-1* from LHB22 could increase VW resistance in F_4_ generation effectively.

**Table 3:** The disease scores in bulked F_4_ families of LHB22×NNG population.

	Disease scores	
Genotype	Flowering stage	Mature stage
Bulk S1	2.85 ^Ab^ ‡	3.16 ^Ab^
Bulk S2	2.98 ^Ab^	3.26 ^Ab^
Bulk R1	1.19 ^Bc^	1.27 ^Bd^
Bulk R2	1.17 ^Bc^	1.34 ^Bcd^
NNG	3.44 ^Aa^	3.50 ^Aa^
LHB22	1.20 ^Bc^	1.39 ^Bcd^
Yu2067	1.22 ^Bc^	1.47 ^Bc^

‡ Different lowercase letter and capital letter in the same column show difference significant at 0.05 and 0.01 level respectively.

## Discussion

### VW resistance to isolate V991

The identification of gene/QTL conferring resistance/tolerance to VW is essential for the development of resistant/tolerant varieties in Upland cotton. The V991 is a prevailing defoliating *V. dahliae* isolate in the Yellow and the Yangtze River cotton growing regions in China [19]. As for the resistance to V991, six resistance QTL have been previously mapped, including three QTL on Chr. 2 and Chr. 16 in an F_2:3_ population from Lumianyan22×Luyuan343 [20], and three QTL on Chr. 9, Chr.17 and Chr.23 in an recombinant inbred lines (RILs) population from Prema×86-1 [19]. These results implied that different germplasms might possess different resistance QTLs for the same VW isolate V991, which was also investigated by VW resistance genes expression analyses [22]–[25]. In the present study, two QTL (*qVW-c6-1*, *qVW-c21-1*) conferring resistance to V991 were identified on Chr. 6 and Chr. 21 explaining 14.44–27.1% of the phenotypic variance and have seldom been reported as controlling VW thus far, which suggests that these two QTL are new QTL resistant to V991. The results of this work offered necessary complement to further understanding of the genetic base of VW resistance to V991 in cotton. These results together provide the possibility of developing new resistant cotton lines by QTL pyramiding through MAS.

### Possible reasons for the differences in QTL results between this research and a previous study [20]

It is necessary to discuss the possible reasons for the differences in QTL results (mentioned in the above paragraph) between this work and a previous research [20] because the resistant parent Lumianyan22 (L22) in that research is the recurrent parent of the LHB22 used as resistant parent in the present study [27]. In that work, based on a genetic map of ten markers on three Chr., three QTL including two (*qVWR-c16-1*, *qVWR-c2-1b*) from L22 were detected at flowering stage in an F_2∶3_ population in artificially infected disease nursery with mixed VW isolates. In the present research, QTL mapping was performed in the LHB22×JM11 population at seedling stage under greenhouse conditions based on a genetic map of 141 markers distributed on 26 chromosomes. And after detecting the two QTL (*qVW-c6-1*, *qVW-c21-1*), we focused on validation of the *qVW-c6-1* in the LHB22×NNG population at flowering and mature stages only in Chr. 6. In other words, QTL detection was not performed on Chr. 2 and 16 and other chromosomes at flowering stage in the present research. We performed QTL mapping at genome level only at seeding stage under greenhouse conditions in LHB22×JM11 population. In addition to the introgression of genetic components from *G. bickii* in LHB22, the differences in disease infection methods, VW isolates used and marker coverage in these two researches might be possible reasons for not detecting those two QTLs (*qVWR-c16-1*, *qVWR-c2-1b*) at seedling stage in the LHB22×JM11 population in the present study, while these factors have been reported to seriously impact VW resistance QTL mapping results in previous researches [7], [16], [26], [40]. For example, some Pima (*G. barbadense*) lines with high VW resistance under naturally infected field conditions were detected as susceptible in VW array under greenhouse conditions [7].

### The stability of QTL and its further use in breeding

The aim of QTL mapping is for MAS, so the stability and facility of QTL were really important. In this study, the major QTL *qVW-c6-1* could be detected in the JM11 background at seedling stage under greenhouse conditions and in the NNG background at flowering and mature stages under artificially infected disease nursery conditions. And the bulked F_4_ experiment indicated the marker-assisted selection of the positive allele of this QTL with two flanking markers was also effective in the NNG background. It should be noted that our present results just indicated this QTL to be effective in these two susceptible variety/line backgrounds, and whether it is effective in other genetic backgrounds need to be tested in further researches. In view of the relative low QTL mapping solution in the present study, MAS using flanking markers on both sides combined with phenotypic screening in a large population was suggested in order to improve selection efficiency once the present results were used to transfer this QTL into other genetic backgrounds. This selection strategy has been proved effective in many researches [41], [42]. In practice, all MAS schemes will be used in the context of the overall breeding program, and this will involve phenotypic selection at various stages [43]. In addition, the development of a RILs population from the LHB22×NNG cross is in progress and it will be applied to evaluate the QTL detected in more environments, to narrow the QTL confidence interval by combining SSR markers and other markers, such as single nucleotide polymorphisms (SNPs), and to detect putative QTLs located on other chromosomes.

## Supporting Information

Figure S1 **The linkage map constructed from the LHB22×JM11 F2∶3 population.** This map contains 141 SSR marker loci on 26 linkage groups assigned to 23 chromosomes, covering 1143.1 cM with an average distance of 8.11 cM between adjacent markers. No markers were mapped on Chr.2, 20 and 25.
(PDF)
