# Supplementary figures and images for: Molecular Mapping and Validation of a Major QTL Conferring Resistance to a Defoliating Isolate of Verticillium Wilt in Cotton (Gossypium hirsutum L.)
Source: PLoS One. 2014 Apr 29;9(4):e96226. doi: 10.1371/journal.pone.0096226 (PMC4004561; doi:10.1371/journal.pone.0096226)

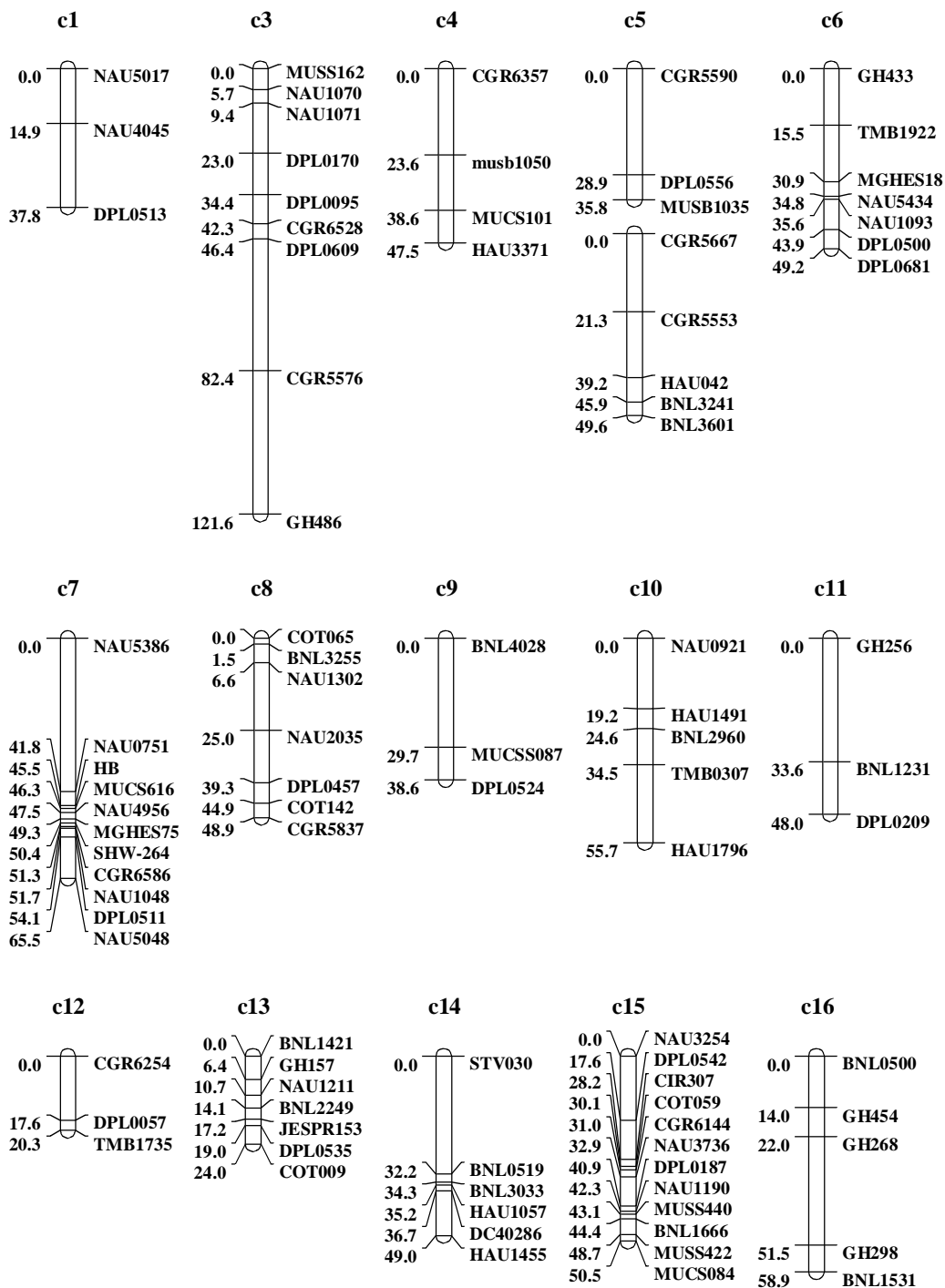

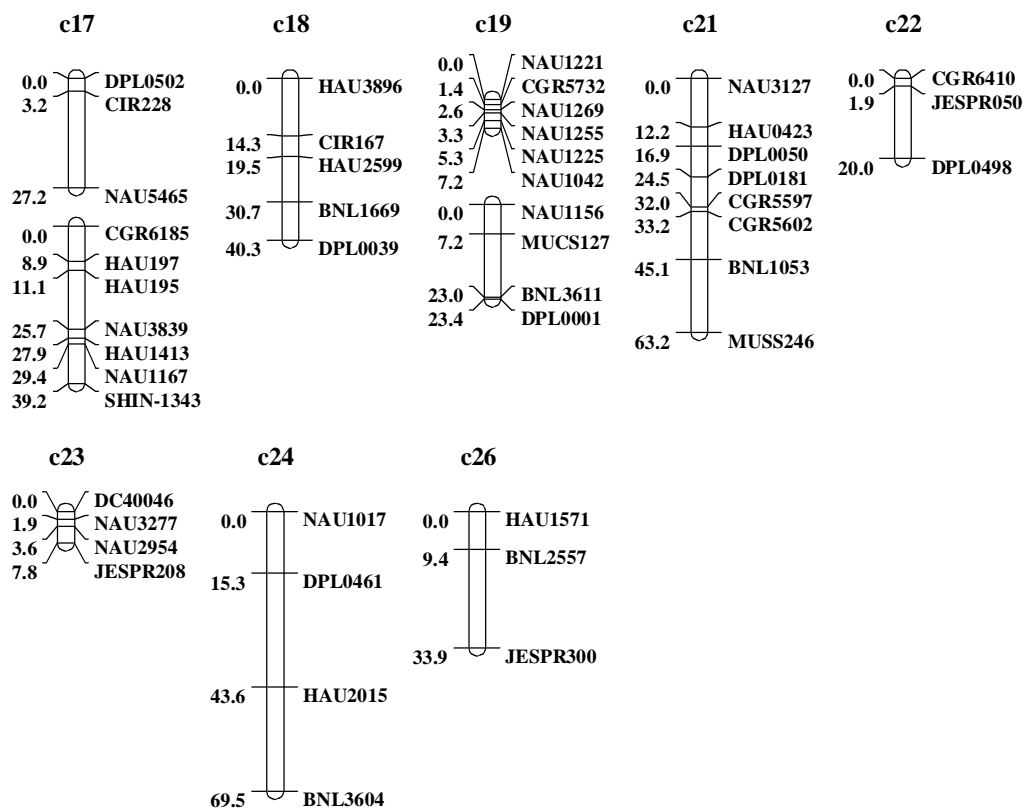

Supplement: Figure S1 — The linkage map constructed from the LHB22×JM11 F2∶3 population. This map contains 141 SSR marker loci on 26 linkage groups assigned to 23 chromosomes, covering 1143.1 cM with an average distance of 8.11 cM between adjacent markers. No markers were mapped on Chr.2, 20 and 25. (PDF) [file pone.0096226.s001.pdf]
